# Translating Neurobehavioral Toxicity Across Species From Zebrafish to Rats to Humans: Implications for Risk Assessment

**DOI:** 10.3389/ftox.2021.629229

**Published:** 2021-02-23

**Authors:** Charles V. Vorhees, Michael T. Williams, Andrew B. Hawkey, Edward D. Levin

**Affiliations:** ^1^Department of Pediatrics, University of Cincinnati College of Medicine, Cincinnati, OH, United States; ^2^Division of Neurology, Cincinnati Children's Research Foundation, Cincinnati, OH, United States; ^3^Department of Psychiatry and Behavioral Sciences, Duke University Medical Center, Durham, NC, United States

**Keywords:** human neurotoxicity, rat neurotoxicity, zebrafish neurotoxicity, developmental neurotoxicity, neurobehavioral toxicity

## Abstract

There is a spectrum of approaches to neurotoxicological science from high-throughput *in vitro* cell-based assays, through a variety of experimental animal models to human epidemiological and clinical studies. Each level of analysis has its own advantages and limitations. Experimental animal models give essential information for neurobehavioral toxicology, providing cause-and-effect information regarding risks of neurobehavioral dysfunction caused by toxicant exposure. Human epidemiological and clinical studies give the closest information to characterizing human risk, but without randomized treatment of subjects to different toxicant doses can only give information about association between toxicant exposure and neurobehavioral impairment. *In vitro* methods give much needed high throughput for many chemicals and mixtures but cannot provide information about toxicant impacts on behavioral function. Crucial to the utility of experimental animal model studies is cross-species translation. This is vital for both risk assessment and mechanistic determination. Interspecies extrapolation is important to characterize from experimental animal models to humans and between different experimental animal models. This article reviews the literature concerning extrapolation of neurobehavioral toxicology from established rat models to humans and from zebrafish a newer experimental model to rats. The functions covered include locomotor activity, emotion, and cognition and the neurotoxicants covered include pesticides, metals, drugs of abuse, flame retardants and polycyclic aromatic hydrocarbons. With more complete understanding of the strengths and limitations of interspecies translation, we can better use animal models to protect humans from neurobehavioral toxicity.

## Introduction

The field of neurotoxicology has grown steadily over the last 50 years as demonstrated by a search on the term neurotoxicity in PubMed. In 1927 only 1 paper cited used the term neurotoxicity, in 1970 that number was 138, whereas in 2019 the number was 3,756. Much has been accomplished but much remains to be discovered. It is useful at this juncture to look at where the field is and ahead to ask how we can develop the field further.

Neurotoxicity may occur in humans, animals, or cell culture. To determine the risk that a chemical is neurotoxic to humans, human data are the most important but also reflect that people have already been harmed. To prevent neurotoxicity in humans, animal data and *in vitro* cell culture data are used to predict and prevent injury. There are advantages and limitations of all levels of analysis. For children, the data come from developmental epidemiological studies. These studies are time-consuming, expensive, and not experimental since subjects are not randomly assigned to groups, have different histories, have experienced influences not relevant to the study, and have complex and uncontrolled exposures that are difficult to identify and measure. These factors lead to interpretations that are rarely definitive. Epidemiological studies establish associations between an exposure and neurobehavioral impairment, but they cannot prove causation or mechanism of action. Generally, multiple epidemiological studies are needed to establish a chemical as a human neurotoxicant. A drawback to human studies is that people were exposed to the agent in question first, which is too late to prevent harm. Furthermore, human development is protracted relative to other species, taking many years to obtain reliable evidence about a potential neurotoxicant. At the other end of the spectrum, neurotoxic effects in older people are difficult to study let alone trace back to a cause if it originated during early development. These characteristics make animal studies advantageous provided they predict human toxicity.

Experimental studies in animals have advantages beyond their shorter life-span: subjects can be randomly assigned to groups, exposure history is controlled, genetic background is more uniform, a range of doses can be tested, invasive procedures related to mode of action are feasible, ADME can be determined, experimenters can be blinded to treatment groups, and sample size can be chosen to fit the study design (Cory-Slechta et al., 2001). Consequently, the risk of neurotoxicity can be determined before damage to people occurs. For these reasons, animal studies are critical for neurotoxicity assessment. However, experimental studies have limitations, too, primarily with respect to cross species extrapolation.

Most of the time, determination of neurotoxicity depends on animal and human data. A problem arises, however, when human data are missing, sparse, or equivocal. *In vitro* data can enhance animal data but can be difficult to extrapolate to intact organisms because cells out of context (their microenvironment) respond differently than in an intact organism (Nebert and Ingelman-Sundberg, 2016), therefore, *in vitro* data are generally not decisive by themselves. Consequently, regulatory authorities throughout the world, rely on animal data to determine the safety of drugs, pesticides, occupational chemicals, and environmental contaminants.

The detection of neurotoxicity is different from other toxicities because of the complexity of the brain (Heyer and Meredith, 2017). This complexity makes identifying the site of damage within the brain and subsequently determining the mode of action for neurotoxic agents challenging. In part this is why neurotoxicity assessments often rely on behavioral evidence along with neuropathological and/or neurochemical evidence (Bolon et al., 2011). Another reason behavioral assessment is used is because it represents the integrated output of the brain and is often a sensitive index of whether a significant effect on brain function has occurred (Piersma et al., 2012; Foster, 2014; Fisher et al., 2019). These factors, together with the fact that most of the human data are behavioral has led to behavior being a major component of developmental neurotoxicity testing (DNT) assessments. Moreover, even if cellular damage is detected, assessing behavior is important since only by measuring behavior can the functional consequences of a cellular effect be determined. Some types of cellular damage induce clear behavioral abnormalities, while others do not change behavior, pointing to the fact that cellular and behavioral effects sometimes do not correlate with one another. Hence, animal data, including behavioral data, are key to hazard identification, dose-response analysis, and overall risk assessment. If unreasonable risk is determined, the chemical becomes the province of risk management.

## Regulatory Neurotoxicity

Protecting people from toxic chemicals originated with U.S. Congressional passage of the Pure Food and Drug Act in 1906 and later the Food, Drug and Cosmetic act of 1938. These statues established the Food and Drug Administration (FDA) and gave it authority to regulate drug safety and efficacy. To implement this authority the FDA required manufacturers of drugs to submit animal data before human trials (Fisher et al., 2019). The Food, Drug and Cosmetic Act was amended in 1962 after thalidomide. This resulted in expanded use of animals to assess toxicity and these guidelines are now standard. For effects on development, they take the form of pre- and postnatal developmental toxicity studies (Bailey et al., 2009), including a newer juvenile toxicity study (Cappon et al., 2009; Piersma et al., 2012; Fisher et al., 2019) for drugs and food additives (Sobotka et al., 1996). These methods are not perfect but have worked well for nearly a century.

For other chemicals, the U.S. Environmental Protection Agency (EPA) was created in 1970. The EPA operates under multiple laws, the two most relevant here are the Federal Insecticide, Fungicide, and Rodenticide Act (FIFRA) and the Toxic Substances Control Act (TSCA). Under these statutes, animal test guidelines were established, including those for adult neurotoxicity (ANT) and DNT. In the EU, guidelines occur through the Organization for Economic Cooperation and Development (OECD) (Makris et al., 2009). From the outset there were advocates for alternatives to animal studies as early as 1959 [Cited in Stephens (2009)]. These evolved into the 3Rs, i.e., replace, reduce, and refine toxicity methods to eliminate and/or minimize animal testing.

The search for alternates to animal testing is an active area of research (Wetmore et al., 2015; Fritsche et al., 2018; Hatherell et al., 2020; Marx-Stoelting et al., 2020; Paparella et al., 2020). However, these approaches are not yet ready for routine use, especially for brain. Recently, the EPA Administrator “mandated” the elimination of animal testing by 2035. However, it is unclear if this is feasible. Much is still unknown about mechanisms of toxicity in general and particularly for neurotoxicity, given the complexities of the brain and even greater complexity of brain development (Heyer and Meredith, 2017). Even if an *in vitro* system for brain became available and even if tested with a set of positive and negative controls (Bal-Price et al., 2018), significant issues would remain, such as, would negative results be trusted or would validation be needed with animals to ensure that a negative was a true negative? What would be done with hits when an *in vitro* screen found evidence of neurotoxicity? Would verification be required using animals? What happens if discrepancies occur between *in vitro* and animal results? How will it be resolved? Would both need to be repeated? What if replication failed to resolve a discrepancy? Will discrepancies be resolved in favor of *in vitro* or *in vivo* results? What will be done about misses, i.e., cases where the *in vitro* screen was negative but human data later uncovered neurotoxicity? Would doubt be cast on the *in vitro* system? Would it be shelved? Would further research be required to determine why it failed? The underlying assumption of *in vitro* screens is that there are a defined number of key molecular events that if altered, adversely affect CNS development and function. Given the complexity of the CNS, how will we know if all important effects are captured by some set of molecular events (adverse outcome pathways or AOPs) (Pistollato et al., 2020)? Who decides when an *in vitro* test system is sufficient to rely on it? How does one know what remains unknown? Would an *in vitro* test battery be used in conjunction with a test system in lower animals, such as zebrafish? These issues have no easy answers and until they are addressed, ANT and DNT tests in animals will be needed.

Species used in neurotoxicological assessment range from invertebrates like c. Elegans and *Drosophila*, to zebrafish, rodents (mice and rats), non-human primates, and people. Each level of analysis has advantages and disadvantages, but the goal is to determine human risk before people are harmed. Therefore, the development of better methods to detect neurotoxicity in animals would provide better protection to people by identifying hazards before they adversely affect humans. Part of improving animal assessments is improving the translation of animal data to humans.

## Zebrafish Neurotoxicity

A species of growing importance for neurobehavioral toxicology is zebrafish. Zebrafish provide an important complementary model intermediate between high throughput *in vitro* cell-based assays and rodent models. There are extensive genetic data available for zebrafish, they have a short life cycle, prolific reproduction, a transparent embryonic stage, a vertebrate brain, and can be tested behaviorally (Bailey et al., 2013). Zebrafish have a clear chorion which permits visualization of neurodevelopmental processes with a variety of reporter systems available (Langheinrich, 2003). There are abundant mutant zebrafish strains, as there are with mice, that can facilitate investigation of gene x environment interactions (Talbot and Hopkins, 2000). Finally, there is a wide variety of behavioral tests that are available to assess neurotoxic impacts on zebrafish behavior including, sensorimotor response, emotion, cognition, and social interaction and are suitable for developmental neurobehavioral toxicology (Bailey et al., 2013). We and others use these behavioral assays to assess the consequences of early developmental exposure to low doses of different toxicants including pesticides, drugs of abuse, flame retardants, heavy metals, and polycyclic aromatic hydrocarbons. For several chemicals, nicotine, chlorpyrifos, diazinon and benzo[a]pyrene, we have assessed the long-lasting effects of early-life exposure to low doses in zebrafish and rats so that direct comparisons can be made.

## Comparative Neurotoxicity: Zebrafish and Rodents

Given the long history of rodent testing in experimental psychology and neuroscience, and the emergence of the zebrafish as a model species, it is no surprise that zebrafish behavioral batteries were developed to compliment the features of similar rodent batteries. Although these tests were adapted to the unique ethology of social fish, several important parallels can be drawn between fish and rodents.

### Exploration and Anxiety-Like Behaviors

Among the most common tests used in these species are tests of activity and anxiety-like behavior, which consist of open-fields or mazes that allow measurement of exploratory behaviors, locomotor activity, and preferences that correspond with anxiety-like or anti-predation functions. Although there is not full consensus on what constitutes anxiety in non-verbal species, anxiety-like behavior generally refers to unconditioned escape or avoidance behaviors which naturally take place in the presence of particular stimuli or environmental conditions, as opposed to the subjective emotional state described by humans. For rats and mice, prototypical tasks include the open-field, elevated plus or zero maze, figure-8 maze, and light-dark test (Takao and Miyakawa, 2006; Walf and Frye, 2007; Bailey and Crawley, 2009). In each case, locomotor activity and exploration are measured through distance moved and transitions between different regions within the test space. Open-fields for fish consist of novel fish tanks (Levin, 2011) and light-dark structures (Stewart et al., 2011; Blaser and Rosemberg, 2012), with constituent behaviors scored in similar ways to their rodent counterparts. Both rodents and zebrafish show spontaneous exploration. Rodents characteristically stay close to exterior walls and enclosed spaces, a pattern known as thigmotaxis, which limits predation risk from X and Y dimensions (i.e., from the sides). Zebrafish show a similar preference for the bottom of a novel tank and reduce their risk of predation in the “Z” dimension (i.e., from above and below). Additionally, both rats and zebrafish show a preference for a dark area over one in the light, again reducing the risk of predation. These varying stimuli and responses can be used to detect neurotoxicant-induced changes in psychomotor and affective processes.

### Fear and Reflexes

Other responses include fear-like and elicited reflex behaviors, such as acoustic startle and responses to aversive or predatory cues. As with anxiety, fear cannot be objectively measured in non-verbal species, so fear-like behavior generally refers to escape or avoidance in the presence of explicitly threatening stimuli, such as electrical shocks, loud noises, or moving objects, and fear-like learning would refer to changes in behavior based on prior interactions with these stimuli. For rats and mice, fear-like and reflexive behaviors are often leveraged to study a variety of responses and learning patterns such as prepulse-inhibition, eyeblink conditioning, and active and/or passive avoidance (Green et al., 2002; Timofeeva et al., 2008; Ohta et al., 2012). With respect to acoustic startle, rodents show characteristic freezing responses, bodily contraction and immobility (Santos et al., 2005), which presumably reduce the detection of the rodent by predators. Higher-order fear-like responses consist of shifts in behavior based on experience with aversive cues, such as foot-shock and loud sounds. Zebrafish have some of the same features, although they present in species-specific ways. Zebrafish are commonly tested for acoustic startle, although their response consists of stereotyped movements rather than freezing, such as the C-start exhibited by larval fish (Lopez-Schier, 2019) or a locomotor spike seen in adult fish (Zeddies and Fay, 2005; Eddins et al., 2010). As with other species, these responses rapidly habituate with repeated presentations. Zebrafish also show fleeing or other anti-predation behaviors in response to cues like fast moving objects, videos of predatory fish, and alarm pheromones (Speedie and Gerlai, 2008; Luca and Gerlai, 2012; Ladu et al., 2015). These stimuli and responses can be used to detect neurotoxicant-induced changes in fear-like and reflexive startle responses, as well as adaptive processes related to these functions.

### Social Behavior

Rats, mice, and zebrafish are all ethologically social species, and have characteristic responses to conspecifics. Rodents have a wide range of sniffing, following, aggressive, and copulatory behaviors (Blanchard et al., 2003; File and Seth, 2003; Kaidanovich-Beilin et al., 2011; Brock et al., 2013) that can demonstrate social interest, reactivity, and engagement. Zebrafish, while less interactive than rodents, are selectively attracted to other zebrafish, and tend to stay in close proximity to related fish, whether physically in the same tank, separated by clear barriers, visible in a mirror, or presented as a video on a screen (Pham et al., 2012; Qin et al., 2014). These varying behaviors can be used to detect toxicant-induced changes in social attraction and engagement.

### Cognition

Among the testing types presented here, the most prominent disparities between neurobehavioral batteries in rodents and zebrafish are on tests of cognitive function. A myriad of cognitive tests exist for rodents covering attention, recognition memory, classical conditioning, operant response learning, navigation, and higher order spatial behaviors, and aspects of cognitive flexibility such as reversal learning and set shifting (Dudchenko, 2004; Bushnell and Strupp, 2009; Tait et al., 2014; Vorhees and Williams, 2014; Braun et al., 2015). Zebrafish exhibit cognitive behaviors as well, although tests are generally limited to lower-level learning and memory, such as reflex-habituation, spatial alternation, conditioned place preference, and basic learning from consequences paradigms, as in the operant three-chamber test (Arthur and Levin, 2001; Levin, 2011; Mathur et al., 2011; Hawkey et al., 2020). This disparity is largely due to practical difficulties in measuring motivated behaviors equivalent to operant chamber lever pressing, foraging, and Morris water maze (MWM)-like behaviors. For example, zebrafish do not manipulate objects as rodents do, but they swim back and forth in a stereotypic, non-goal directed manner, and are highly susceptible to stress from repeated handling. Additionally, zebrafish cannot be reliably identified as individuals, and so cannot complete multiple days of testing as rodents can. Methods development are underway to create tasks that work around these obstacles, but at present, these data remain limited.

## Developmental Neurotoxicity in Zebrafish vs. Rats

Zebrafish provide unique opportunities to investigate neurotoxicity, although their broader benefit depends on their ability to predict and/or complement similar studies conducted with other model systems, particularly in rats and mice. We have conducted studies of the persistent behavioral effects of a variety of chemicals on behavioral function in zebrafish and rats, including nicotine, chlorpyrifos (CPF), diazinon (DZN), and benzo[a]pyrene (BAP), and a number of labs have investigated these compounds in one or the other of these species. We review the effects of these compounds below, with the intention of highlighting the degree to which compounds with established neurotoxicity in mammals also produce behaviorally relevant disruptions in zebrafish. Other chemicals including ethanol, lead, and mercury were assessed in both zebrafish and rats by others. A summary of these comparative effects is also included.

## Translating Zebrafish Effects to Rats

### Nicotine

Nicotine, the primary psychoactive chemical in tobacco, has been shown in a variety of studies including our own to cause persisting behavioral dysfunction after early developmental exposure. Zebrafish with embryonic nicotine exposure exhibit changes in larval locomotor, photomotor, and startle functions (Parker and Connaughton, 2007; Ali et al., 2012; Gauthier et al., 2020; Holden et al., 2020), as well as persistent changes in startle responsivity in adulthood (Eddins et al., 2010). It was also noted that chronic exposure to nicotine later in development leads to persistent changes in neurobehavioral function, including anxiety-like responses in a novel tank and shoaling (Stewart et al., 2015). Correspondingly, we find that rats with fetal exposure to nicotine have a range of neurobehavioral effects, including hyperactivity, increased nicotine self-administration, and impairments in attention and memory [e.g., (Cutler et al., 1996; Levin et al., 2006; Hall et al., 2016; Hawkey et al., 2019)]. These data also complement a broader rodent literature that show early exposure to nicotine leads to lasting changes in brain and behavior [e.g., (Ernst et al., 2001; Vaglenova et al., 2004; Schneider et al., 2011)]. Using these two models, it is apparent that developmental exposure to nicotine is neurotoxic in both species, likely due to strongly conserved cholinergic mechanisms.

### Chlorpyrifos

CPF is a widely used organophosphate insecticide that causes significant long-term behavioral effects in rats and zebrafish. Zebrafish with embryonic CPF exposure exhibit changes in larval locomotor and motor functions (Levin et al., 2004; Tilton et al., 2011; Yang et al., 2011; Padilla et al., 2012) and persistent behavioral effects in adulthood, including hyperactivity, enhanced startle responses, altered response latencies, and impaired operant learning (Levin et al., 2003; Eddins et al., 2010; Sledge et al., 2011). In rats, fetal exposure to CPF is associated with a range of neurobehavioral effects, including altered locomotor habituation trends, impaired spatial alternation, and radial arm maze performance [e.g., (Levin et al., 2002; Icenogle et al., 2004)]. We similarly found neonatal CPF exposure leads to long lasting behavioral effects, including locomotor habituation trends, open arm preference in the elevated plus maze, and increased error rates in the radial arm maze (Levin et al., 2001; Aldridge et al., 2005). Other labs similarly showed behavioral changes in mice following CPF exposure [e.g., (Ricceri et al., 2003, 2006; Venerosi et al., 2006)]. Taken together, rodent and fish work suggests that developmental exposure to CPF is broadly neurotoxic and can impact many aspects of behavioral function in both species. This complements the previously discussed effects of nicotine in both species, as organophosphates can also influence cholinergic function during development, albeit through different mechanisms.

### Diazinon

DZN is another organophosphate insecticide that continues to be widely used after the curtailment of chlorpyrifos use. Zebrafish with embryonic DZN exposure exhibit changes in larval locomotor behavior (Padilla et al., 2012; Cao et al., 2018), as well as hyperactivity and reduced anxiety-like diving in the novel tank in adulthood (Bailey et al., 2013). Rats with prenatal or postnatal DZN exposure exhibit a wide variety of behavioral effects, including alterations in multiple affective behaviors, hyperactivity, impaired spatial alteration, recognition memory, prepulse inhibition, and working memory errors in the radial arm maze (Roegge et al., 2008; Timofeeva et al., 2008; Hawkey et al., 2020). As with CPF, developmental exposure to DZN is neurotoxic in both species and can lead to persistent changes in behavior.

### Benzo[a]pyrene

Benzo[a]pyrene (BAP) is a common polycyclic aromatic hydrocarbon pyrolysis product widely distributed in the environment. Zebrafish with embryonic BAP exposure exhibit changes in larval locomotor behavior, as well as deficits in learning in adulthood (Knecht et al., 2017). Rats with fetal exposure to BAP have lasting neurobehavioral effects, including hyperactivity in adolescence and adulthood and disruptions of typical sex-differences in novel-environment suppressed feeding (Hawkey et al., 2019). Other labs similarly show persistent behavioral changes following early BAP exposure in rats or mice, including deficits in the righting reflex, hyperactivity, enhanced open arm time in the elevated plus maze, and impairments in learning tests like the Y-maze and MWM (Chen et al., 2012a; Zhang et al., 2016). These studies indicate that BAP is neurotoxic in both species and can impact several aspects of behavior, while acting through quite different mechanisms (e.g., aryl-hydrocarbon receptor) than the previously mentioned insecticides.

### Ethanol

Developmental ethanol exposure produces one of the most well-characterized syndromes of developmental neurotoxicity, the fetal alcohol syndrome. Developmental ethanol exposures are associated with a diverse array of neurobehavioral outcomes, including hyperactivity, affective symptoms, and cognitive or learning deficits. This is seen in humans (Mattson et al., 2019), rodent models (Barron et al., 2016), and zebrafish (Fernandes et al., 2018). In collaboration with Cole et al. (Bailey et al., 2015), we found that a brief embryonic exposure to ethanol produces long-term behavioral impairments in zebrafish, including hyperactivity and reduced anxiety-like behaviors. Others found complementary long-term effects, such as reduced anxiety-like diving in the novel tank, hyperactivity, and shoaling deficits [e.g., (Fernandes and Gerlai, 2009; Baiamonte et al., 2016; Burton et al., 2017)]. Ethanol exerts developmental neurotoxicity through several mechanisms, including direct modulation of excitatory and inhibitory activity across the brain, and its risk to the neurobehavioral function is readily detected across these model species.

### Metals

Another major area of study are neurotoxic metals, including lead, cadmium, and methylmercury. Like ethanol, developmental exposures to these compounds are associated with a range of developmental, behavioral, and cognitive symptoms in humans (Sanders et al., 2015; Karri et al., 2016; Caito and Aschner, 2017), and corresponding deficits in rodents [e.g., (Lucena et al., 2010; Abu-Taweel et al., 2013; Weston et al., 2014; Basha and Reddy, 2015)] and zebrafish (Lee and Freeman, 2014). In zebrafish, metals produce multiple effects. For example, Tu et al. (Tu et al., 2017) reported that lead, cadmium, and manganese dose-dependently produced locomotor hypoactivity in larvae with embryonic exposure. Further, early exposure to lead leads to disruptions in startle (Rice et al., 2011), social (Weber and Ghorai, 2013), and learning functions in zebrafish (Chen et al., 2012b). Similarly, exposure to methylmercury can lead to disruptions in swimming and learning or memory in a T-maze (Strungaru et al., 2018) and spatial alternation (Smith et al., 2010). Metals can interfere with a variety of intracellular processes, and their influence on behavioral functions later in life are readily detected across these model species.

### Predictive Validity of Zebrafish Models

As zebrafish are an economical model for screening of potentially neurotoxic compounds, there is a natural interest in using zebrafish as an intermediate model to identify which candidates should be prioritized in future rodent work. However, such a decision requires a robust predictive relationship between zebrafish and mammalian models. At minimum, it must be reasonable to predict that compounds which are toxic in the zebrafish nervous system are also toxic in the mammalian nervous system. The studies reviewed above generally support this claim, as toxicants with varying properties and mechanisms of action produce behaviorally relevant disruptions in both zebrafish and rodents. Ideally, the exact manifestations of those disruptions would match up between the two, in much the same way that the tests designed to analyze zebrafish behavior were designed to model behavioral functions in rodents. However, as evidenced by the review above, this is not necessarily the case. So, profiles of impairment observed in zebrafish should be interpreted with care and within a framework that is sensitive to the specific neurobiological and behavioral context of the animal. In short, attempts to translate neurobehavioral toxicity from fish to mammals should focus on aspects of these analyses which have strong predictive validity, such as the relative vulnerability of the nervous system to a toxicant, relevance of underlying changes to behavioral outputs, and well-conserved mechanisms of toxicity.

## Summary of Zebrafish as a Model

Zebrafish are a complementary model species that can allow hypotheses about the potential toxicity of a compound to be tested in a setup which preserves many advantages offered by both higher and lower models of toxicity. For example, zebrafish offer an intact, vertebrate nervous system and behavioral repertoire which cannot be matched by *in vitro* techniques, while providing practical, cost and throughput advantages over mammalian models like rats. At present, there are many thousands of chemicals which lack developmental toxicity testing, and this backlog cannot reasonably be addressed using rodents alone, so there are many incentives for including zebrafish in the arsenal of techniques. However, it is important to be clear about their placement within the hierarchy of available models and their applicability to human health concerns following developmental chemical exposures.

As vertebrates, zebrafish share many genetic, developmental, neural, pharmacological, and behavioral attributes with humans and other mammals. However, notable differences exist which affect the translation of zebrafish data to mammalian toxicity.

One of the principal points of concern is for sex differences in neurotoxic response. Sex differences in toxicant vulnerability are present in the rodent literature and to a certain degree within zebrafish studies as well. However, sex characteristics are determined quite differently in teleost fish than in mammals, so we and others have been careful not to interpret sex differences in zebrafish as predictors of human sex differences in vulnerability.

Another difference between these species concerns brain architecture. The predominant covering layer in zebrafish brain is the optic tectum whereas in the brain of rats as with all mammals it is the neocortex. Zebrafish have little telencephalon with the main structure being the midbrain, whereas the midbrain in the rat is relatively small. Surprisingly, there is a degree of confluence of neurotoxic effects in zebrafish and rats. This may have to do with similarities in the cellular and synaptic structure of the two species. If the principal target of a neurotoxic agent is cellular and synaptic rather than being dependent on brain region that agent should have similar effects in zebrafish and rats. In contrast, if a brain region is targeted and that region has different presence in zebrafish then that agent would be predicted to have different expression in the two species. Specification of a conserved neurobiological mechanism, such as a cellular structure, protein, or neurotransmitter, is then critical for a detailed translation of zebrafish toxicity to mammals.

An additional difference is in physiology and genetics. For example, the ectothermic nature of zebrafish allows temperature to regulate physiology, development, biological rhythms and even toxicity to a substantial degree. These effects may be influential within this model, but do not have obvious homologs in mammals. Additionally, an estimated 70% of zebrafish genes are homologous with mammalian genes, which provides a wide array of translatable mechanistic pathways and mechanisms of action within neurodevelopment and toxicity, but also provides non-homologous genes whose influence is not directly relevant to humans. This is complicated further by the genome duplication that is characteristic of teleost fish. As with brain structure, molecular mediators of toxicity must be investigated with attention to well-conserved mechanisms of risk, rather than assuming that, all elements necessarily translate to mammals.

A final consideration is pharmacokinetics, which influences the translation of a particular chemical exposure protocol from one species to another. Rats and other mammals are generally exposed to toxicants through their diet or through manual administration (i.e., gavage, injection), each of which has its own impact on dose, absorption, and metabolism. During pregnancy, the placenta acts as a secondary barrier which influences the pharmacokinetic distribution to fetal tissues. Zebrafish, by contrast, are immersed in a toxicant-laced medium, and absorb it directly from the media and/or through a porous chorion which surrounds the embryo during the earliest portions of development. These differences influence the distribution of the toxicant to the animal and make it difficult, though not impossible, to generate rat-equivalent doses of a toxicant of interest.

In summary, zebrafish offer a valuable complement to rats for assessing behavioral toxicity. They can be very useful for screening many chemicals and mixtures in preparation for follow-up studies in rodent models. The behavioral test batteries developed for zebrafish provide analogous assessments to rodent sensorimotor, emotional, and cognitive functions. In addition, zebrafish can provide valuable mechanistic information concerning the neurodevelopmental processes affected by toxicant exposure that lead to behavioral dysfunction. In providing a basis for rodent studies, it is important that the unique biology and behavioral repertoire of the zebrafish is taken into account and that relevant mechanisms of toxicity are sufficiently conserved to support the predictive validity of the model.

## Translating From Rats to Humans

Rodent neurotoxicological research relies on the investigator's knowledge of animal behavior, brain anatomy, and physiology. Investigators use methods they know to test chemicals and drugs they suspect may have adverse effects. All known developmental neurotoxicants were found through curiosity-driven experimentation of this kind. This was the case for lead, methylmercury, PCBs, manganese, insecticides, cocaine, methamphetamine, isotretinoin, fenfluramine, antiepileptics, fluoride, tetrachloroethylene, chlorpyrifos, brominated diphenyl ethers and others (Grandjean and Landrigan, 2006, 2014; Vorhees et al., 2018). Despite using varied methods, data accumulated to the point that animal and human data were sufficient for regulatory action to be taken. Once a developmental neurotoxicant is identified, its regulation depends on the type of chemical it is. For drugs, the FDA has a variety of options. They can decline to approve it if it is a new drug that exhibits developmental neurotoxicity in preclinical experiments or approve it with warnings about potential developmental neurotoxicity if it is intended for use in adults, or if such effects are found after approval, the drug may be withdrawn from the market. For illicit drugs that pose neurotoxicity risks in addition to abuse liability, law enforcement and public health warnings are used. For pesticides and other chemicals, animal and/or *in vitro* tests are required that may trigger the need for DNT. If developmental neurotoxicity is identified, it becomes the province of EPA risk management. Historically, most of the known developmental neurotoxicants have long-term epidemiological studies showing adverse effects in children and animal data before regulatory actions were taken. If animal and *in vitro* methods were to be improved, however, adverse outcomes in children could be prevented rather than reacted to after the fact.

For animal studies, the process of identifying developmental neurotoxicants has not been helped by the broad range of methods used in different laboratories. One may ask, is this the most effective approach. Or would a few standardized tests used across labs be helpful? Is the current unstandardized lab to lab variation in methods even a problem? One piece of evidence that it is, may be found in regulatory guidelines from EPA and OECD. After 40 years, these agencies remain uncertain what tests to recommend, unlike in other areas of toxicity assessment. The field is stuck on the issue of whether guidelines should be proscriptive, generic, or somewhere in between, such as outlining test features without proscribing specific tests. Should methods be left to the investigator to use their scientific judgment or mandated? At present, most guidelines leave much leeway to the investigator. The result is a body of data using tests of unclear sensitivity, reliability, reproducibility, validity, and no inter-laboratory comparability.

An issue when it comes to behavior is the large range of tests and testing protocols available. This is evident in the literature and why the field can be difficult to sort out because one can find studies on the same chemical resulting in behavioral changes that are different across studies or even opposite from one another. Is it time to change the approach and specify a core set of more standard methods but still leave room for investigator judgment? This is not intended for basic research, only for regulatory studies. Academic labs should always be open to innovation, but regulatory studies need to emphasize sensitivity, reliability, reproducibility, and validity using methods that translate to humans as much as possible. Given the goal of protecting human health, it follows that the starting point for regulatory studies should be to make use of existing human data as the basis for choosing best fit-to-purpose animal methods (Lanzoni et al., 2019). This was not possible 50 years ago, but it is feasible today because there is a significant literature on human developmental neurotoxicants. These data can and should be used to select animal methods that tap homologous functions in both species and incorporate them into guideline studies. Working backwards from the human data is it possible to identify animal tests that reflect the same underlying processes that are affected in children? Is this possible given how different rodents are from people? We suggest it is eminently possible. For example, in humans there are different kinds of memory mediated by different brain regions (Buzsaki and Moser, 2013; Ferbinteanu, 2020), and these relationships are well-characterized. For instance, there is working or short-term memory and reference or long-term memory. Reference memory consists of explicit and implicit memory. Explicit memory consists of episodic, spatial, and declarative memory. Implicit memory consists of egocentric/procedural and stimulus-response memory. Egocentric memory consists of path integration and route-based memory.

Are the structures and functions comparable in both species? Within limits, they are. Rats have homologous forms of memory as people. Rats have working and reference memory. Reference recall in rats consists of explicit and implicit memory as in humans. Rats have episodic and aspects of declarative memory as well. In people declarative memory is for people, places, things, and events. Rats do not have memory for people obviously, but they do for things, events, and places. Therefore, if a chemical impairs memory for places in children, then the inference is that this type of memory will be affected in rats, and more importantly the converse is highly likely, if place/spatial memory is impaired in rats, it will be affected in children, a testable hypothesis, although ideally it will never be tested if the animal data prevent children from being exposed.

Comparisons such as this are based on research on brain-behavior relationships from neuroscience. The hippocampus and surrounding structures (subiculum, entorhinal cortex, perirhinal cortex) are areas that mediate spatial navigation and recognition memory in rats, non-human primates, and people. Not only is there a correspondence between these structures across species, test methods developed in rats have been adapted to humans (Cornwell et al., 2008; Brown et al., 2014, 2016). For example, the MWM developed for rats was modified to a virtual spatial maze for people that in combination with fMRI show homologous regions activated in both species during navigation (Brown et al., 2014). Adaptation of other tests exist for working and egocentric memory (Baumann and Mattingley, 2010; Cullen and Taube, 2017). In rats, working memory may be assessed with methods such as the radial-arm maze. In people, a method that assesses working memory looks different but relies on the same concept, such as digit span and letter-number sequencing. In both species these tasks activate the hippocampus and prefrontal cortex (PFC). In rats path finding can be assessed using the Cincinnati water maze (CWM) and in people using virtual city-scape maps. In both species these tasks activate the neostriatum (Jog et al., 1999; Hartley et al., 2003; DeCoteau et al., 2004; Botreau and Gisquet-Verrier, 2010; Delcasso et al., 2014). In combination with such behavioral evidence there are electrophysiological and neuropharmacological data in rodents that corroborate these relationships. These relationships form the foundation for animal tests that better correspond to those human brain functions identified in children after exposure to developmental neurotoxic agents.

Epidemiological data show that memory is affected in children from a number of developmental neurotoxicants (Grandjean and Landrigan, 2006, 2014; Vorhees et al., 2018), therefore, the correspondence between human findings and homologous brain structure-function tests in rats is a sound basis for better assessment and will provide leads for where to search for cellular effects. Unfortunately, most of the human epidemiological and animal behavioral data are disconnected. Importantly, the most frequently identified effects in children are for higher cognitive functions. They are not for urination frequency, salivation, ear twitch, whisker movement, paw placement, reaching, edge avoidance, muscle tone, foot splay, etc. Nor for surface righting, inclined plane, pivoting, bedding orientation, swimming ontogeny, wire hanging, auditory startle emergence, etc. Rather the effects are on complex forebrain functions. Most of the human effects are on learning, memory, attention, and executive functions. Rats can perform executive functions, sometimes in mazes, or using schedule-controlled operant conditioning. With operant methods, after training, rats can be put on complex schedules of reinforcement and assessed for discrimination, delayed matching, and delayed non-matching to sample, differential reinforcement of low rates of responding (DRL), vigilance, signal detection, delayed discounting, and others. However, these methods come with a cost. They require extensive training and extended testing. Moreover, there can be large inter-individual differences in performance that make analyzing group data using these methods challenging. Water mazes cannot assess all these functions, but for those they can assess they are much more efficient than operant methods.

## Human Developmental Neurotoxicity Findings

To see what functions are affected in children exposed to neurotoxic chemicals a literature review was undertaken (Vorhees et al., 2018). The literature review was not intended to be exhaustive but rather to identify the major behavioral effects for the best-known developmental neurotoxicants. The compounds were lead, methylmercury, manganese, pesticides, PCBs, PBDEs, bisphenol A, airborne particulates, cocaine, alcohol, marijuana, methamphetamine, and nicotine. For these compounds, the most frequently identified effects were cognitive. They include effects on memory, attention, spatial processing, learning, executive function, IQ, and increased rates of attention deficit hyperactivity disorder (ADHD). Less frequently effects were found on motor coordination, anxiety, and social function/cognition. Can rodents, model all these functions? Within limits, yes. To the extent this has not been done so far can be traced to how basic research labs operate compared with contract labs. Contract labs have more capacity than academic labs to test large numbers of animals. Up until now contract labs have not focused on rat-to-human comparability. Consequently, we find the field divided. At one end, basic research labs focus on discovery using sensitive tests that map to human traits using methods they often develop and at the other end, contract labs focus on standardized methods not based on brain structure-function relationships. We suggest that in a regulatory context a better way is to incorporate methods from neuroscience into contract lab studies with brain structure-function comparability as the guide to which tests to select. Some standardization might benefit academic laboratory comparisons as well. This change could strengthen cross-species comparisons and increase the reliability and validity of animal testing at predicting human developmental effects.

## Recommendations

In the following, the authors offer suggestions on how regulatory DNT studies might be improved. The purpose is to stimulate discussion of how best to restructure DNT studies, so they better reflect the human data using brain-behavior relationships as a means for selecting rodent methods that parallel the domains most affected in children. However, what follows is not intended to be prescriptive but to stimulate discussion.

What tests have sufficient data to recommend them to meet this objective? In considering this there are practical and theoretical issues. The approach we suggest is not to pile on more and more tests. To do so runs the risk of making a DNT battery that is cumbersome, expensive, and impractical. Rather we argue for the selective rescission of some methods, streamlining test ages, and adding a few carefully chosen evidence-driven tests that provide better animal to human comparability.

As noted, epidemiological studies find most of the effects in children from neurotoxic exposures are on working memory, spatial memory, procedural memory, attention, i.e., ADHD-like behavior, and IQ. Tests suitable for assessing these functions in rats are numerous and include for working memory tests such as the radial-arm maze (RAM: appetitive or water), spontaneous alternation (Y- or T-maze), novel object recognition (configured for short-term retention), and contextual fear conditioning (which also taps amygdala function). For spatial learning and memory, tests include the MWM, the Barnes maze, some T-maze methods (Ferbinteanu, 2020), and novel object recognition (configured for long-term memory). For procedural/egocentric learning and memory, tests include the Cincinnati water maze (CWM) (Vorhees and Williams, 2016), Whishaw retrieval test (Whishaw and Maaswinkel, 1998), proximal cue water mazes, and cued T-mazes (Ferbinteanu, 2020).

Tests that might be reduced from the EPA DNT would be those assessing rudimentary functions, such as observational methods that rely on subjective ratings for attributes with no relationship to findings in children and have unknown neural substrates (Cory-Slechta et al., 2001). Automated open-field locomotor activity should be kept but reduced to one or two test ages. Acoustic startle should be kept but changed to include prepulse inhibition of startle to obtain more information for essentially the same test time it takes to run startle habituation and reduce the number of test ages. Other than removing observational methods, most of the streamlining would be through reducing some of the pre- and peri-weaning testing. What is the basis for this recommendation?

What is the value of early assessment in a DNT regulatory context? If one is modeling children, early assessment may be viewed as modeling child development. There is no question that early testing in children is valuable. The question is whether that value translates equally to animals. There are limitations to the comparison between rats and humans for early assessments. First, an early effect in animals may be transient, and not an enduring effect. If it is a transient delay that returns to normal without consequence, then it has no probative value; in fact, it is counterproductive because it uses resources that could be used elsewhere. If the delay continues and is present in adults, then it added nothing to hazard identification since it occurred in the animals as adults. Second, since DNT exposure is pre- and postnatal up to weaning, preweaning testing is done while the compound is on-board. This makes it difficult to determine if effects are acute or enduring. Since the goal of regulatory DNT testing is to determine if there are long-term consequences, effects from concurrent compound exposure complicates interpretation.

Why are early tests done in developmental epidemiological studies? Much of the data on the detrimental effects of lead, for example, were obtained from prospective studies of children and these data were of great value because they were uncovered long before the children reached adulthood. Because of this steps were taken to restrict and later eliminate consumer exposure to lead. Lead adversely affects child development at all stages but in different ways and therefore different age-appropriate tests and interventions were undertaken. But it takes decades to conduct longitudinal studies in children, and it would be a lost opportunity not to evaluate them until they are adults. Second, cognitive abilities change over the course of development. Different abilities appear at different ages and some require later stages of brain maturity before they can be assessed. There are cognitive abilities that children (and young rats) cannot master until later in life, testing for that ability before it is developed is ineffective. While children can learn more than once thought, some of the tests developed to ascertain these early capacities have no homologous method in rodents. The FDA supports the view that early testing with drug on-board is not relevant to risk assessment when it comes to determining long-term neurotoxicity. If an early effect is found from a new drug but disappears later, little weight is placed on it. If an effect is not found early but appears later, it is a concern. If effects are found early and late, the late effect is used for risk determination.

Why does the EPA DNT require multiple early test ages and one when the animals are adults? Perhaps it is left-over from when the field expected that early tests would predict adult outcomes. However, this is not what decades of studies using preweaning testing found. As a result, few academic laboratories use the rudimentary early tests, such as surface righting, air righting, inclined plane, cliff avoidance, pivoting, coordinated walking development, auditory startle emergence, etc., because these tests proved to be poor predictors of later outcome.

Several tests of learning and memory were mentioned above, leading to the question of, how well do young rats learn tests such as the MWM, CWM, or RAM? Young rats can learn the MWM if the pool is small and the platform large, but even then the learning curve is often shallow if the rats are under 25 days of age (P25) (Schenk, 1985; Rudy et al., 1987; Rudy and Paylor, 1988; Tonkiss et al., 1992; Carman et al., 2002; Vorhees and Williams, 2014). Since spatial difficulty in a search task is a function of search area relative to target area it is easy to configure a maze with a small surface to platform ratio that young rats can learn, but the question becomes, what strategy are they using, is it spatial or non-spatial? There are data showing that when the ratio of pool to platform area is small, rats use non-spatial strategies (Braun et al., 2015). This can become a problem if rats use a non-spatial strategy and the experimenter does not realize it and mistakenly interprets it a spatial deficit. It is safer to use mazes that are large enough to avoid such problems. Rats become proficient at spatial searching tasks by P50. Pool diameters for adult rats of 183 cm diameter are suitable, but diameters 200–213 are preferable, and larger ones of 244 cm are even more sensitive to spatial impairments in rats with 10 cm diameter or smaller platforms; pool sizes of 150 cm and smaller in rats, while they show learning, are subject to non-spatial strategies (Schenk, 1985).

For the CWM, when run in complete darkness to prevent use of distal cues thereby forcing rats to use internal (egocentric) cues, cannot be learned before P40. Even when the maze is simplified to 5 or 6 multiple Ts rather than 9–10, young rats find the task difficult and some never learn it. This test works best at P50 or older.

The change in perspective we suggest is overdue in regulatory studies. The outmoded idea that if a test battery has “a” test, any test, of learning and memory then that is sufficient to cover learning and memory is incorrect. This view is slowly changing but it remains in some quarters including by some regulatory bodies. Many neurotoxicity test batteries have a single test of learning and memory. Sometimes the same test is given twice or a simpler one is used in young animals and somewhat more challenging one in adults. Discussions about EPA DNT guidelines have not addressed this issue. We suggest that three tests, one for each type of learning and memory is needed and well-justified by decades of neuroscience research.

The future of *in vivo* rodent DNT guidelines should scale-back current preweaning tests. They are less productive, subjective, and not predictive. Testing should start at P50-60 with the following tests: (1) automated open-field locomotor activity, (2) startle with prepulse inhibition (acoustic or tactile pulses with acoustic or light prepulses), (3) a test of working memory, (4) a test of spatial learning and reference memory, (5) a test of egocentric/procedural learning, and (6) a test of attention; the latter may be part of one of the tests of learning and memory. The requirement to include males and females balanced by litter is essential and all data should be analyzed with sex as a factor, not by separate analyses on each sex.

For these types of memory systems, we suggest a RAM (appetitive or water) for working memory, or spontaneous alternation, short retention interval novel object recognition, or MWM using a matching to sample method, but not passive avoidance. The key to a good working memory test is that responses are trial-dependent, i.e., after a choice, the next choice depends on memory of the one preceding it. For mazes there are advantages of swimming tasks compared with those that are appetitive (Kant et al., 1988). For spatial learning/reference memory we suggest the MWM with a probe trial 24 h after the last platform trial to test reference memory. We favor conducting the test in phases to assess spatial navigation and cognitive flexibility (an executive function) because there are data showing that second and third phase procedures can unmask effects that may not be seen in the first phase (acquisition) (Pitzer et al., 2019). If one sets up the MWM, then with a little additional effort much more data can be gained by these subsequent phases. The phases we recommend are acquisition, reversal (platform in the opposite quadrant), shift (platform in a third, adjacent quadrant), and cued-random. The latter is a control procedure to ensure that rats see and can navigate directly to a visible platform when distal cues are blocked from view. For egocentric learning, we currently use the 10-T CWM, but we used the 9-T version for many years, and it works well. The CWM is best run in the dark. Rats are given 2 trials/day for 18–24 days. But before maze trials, it is necessary to train rats the day before by giving them 4 trials in a straight swimming channel (e.g., 15 × 244 cm). This can be run in the light with a submerged platform at one end and the start at the opposite end. Straight channel trials teach rats how to escape which is essential before they enter the maze. By timing latency to traverse the length of the channel, data are captured that reflect whether all groups are equal in swimming ability and motivation to escape. Without this training, failure rates in the CWM are high, which defeats the purpose of the test. When setting up the CWM, it is crucial that control rats be tested in pilot experiments to ensure they can learn it before running a major experiment. If failure rates among controls do not decline after 9 days, then something is amiss in how the maze is setup. In that case, it is advisable to seek advice. Alternatively, appetitive T-mazes can be used but only if appropriate protocols are used (Ferbinteanu, 2016).

Why do we recommend water mazes? Rats are natural swimmers and unlike mice will search assiduously, whereas mice often display off-task behaviors. Mice may float, display thigmotaxis, mount the platform then jump off, touch the platform then swim away, get on the platform, and jump toward the edge of the pool, fall back in the water, and start searching again (and should be removed immediately). Rats rarely do these things, but water escape is motivating, requires minimal (CWM) or no (MWM) training, unlike appetitive tasks that can require extensive training (Delis et al., 2016), and if configured properly rodents have excellent learning curves with intermediate slopes reflecting that the task is in the optimal, not-to-hard, not-to-easy, range. If properly conducted, all control rats reach proficiency in a few days (5–6 with 4 trials/day for the MWM, 2 days for the RWM, and 18 days for the CWM, 2 trials/day in the dark or 5 days when tested in the light). The only concerns expressed against water mazes is that they are stressful. While correct, so are appetitive tasks. Forcing rats to lose 15% of their body weight by food deprivation is also stressful. Foot-shock based tests, such as active and passive avoidance tests, are even more stressful but for some reason remain in widespread use without criticism, while water mazes that are less stressful than shock are criticized around stress. This view contradicts the evidence and is not credible. When it comes to swimming, the fact is that rats habituate to the swimming experience, and the stress it causes in the beginning, based on corticosterone measurements taken after removal from a MWM (Skelton et al., 2007), declines. Across test days, MWM performance improves, escape paths become more direct and efficient and trial times decline. An important factor mitigating stress in water mazes is the fact that locus of control is with the animal. As rats learn to escape, they become more efficient at swimming directly to the goal. This contrasts with the forced swim test (FST) where the task is designed to be stressful by placing the animal in a container from which no escape is possible. Removal of locus of control induces defeat or “behavioral despair” (Porsolt et al., 1978). It is confinement with no possibility of escape that induces the high corticosterone levels, not swimming to an escape platform. It may be that some concerns about water mazes stem from association with the FST, a test designed to induce stress that is not germane to solvable water mazes. Given that water mazes are not highly stressful, are sensitive, reliable, and efficient, they merit further consideration in regulatory testing guidelines.

For the CWM, it is ideally run in the dark. It is a multiple T-maze with 9 or 10 decision points, with each branch being a T-shaped cul-de-sac. It is a task of sufficient difficulty that some control rats reach the 2-min trial limit for the first 4–5 days. However, once the goal is found, performance improves steadily. The CWM is one of the few tasks able to differentiate striatal from hippocampal effects. Effective learning in the CWM is dopamine-dependent. We have tested compounds that impair performance in the MWM but not the CWM and vice versa or that cause more severe deficits in one compared with the other. Absolute separation is uncommon, but disproportionate effects are often seen, and there are reciprocal connections between the striatum and hippocampus that presumably account for such overlap (Ferbinteanu, 2020). We find this in rats with 6-hydroxydopamine striatal lesions. These lesions severely impair CWM learning but cause effects on MWM learning as well (Braun et al., 2012, 2015, 2016). In genetically engineered knock-out rats, in which *latrophilin-3* is disrupted (the LPHN3 protein is expressed primarily in striatum and to a lesser degree in hippocampus) severe learning deficits are observed in the CWM that are more severe than in the MWM (Regan et al., 2020). Differences between the mazes are also found with drugs that deplete striatal dopamine more than 5-HT vs. those that deplete 5-HT more than dopamine. Dopamine-depleting drugs produce greater CWM than MWM impairments whereas predominantly 5-HT depleting drugs affect MWM performance more than CWM performance (Vorhees et al., 2011). That different water mazes measure different types of learning and memory mediated by different brain regions is an advantage that could be exploited to if used in regulatory studies.

When it comes to other tests, concerns arise about some of them. These include the water M-maze (and variants) and passive avoidance. The problem with M or other two-choice water mazes is that rats have strong side preferences, stronger than seen in dry T-mazes. Even if reinforced to the non-preferred side the turning bias is not neutralized and leads to errors of habit rather than from the process of learning. A second problem is that the cost of an error is too low in two-choice water mazes, such that no matter how many times a rat chooses the incorrect arm, it can correct the error quickly with no significant consequence from the mistake. Confinement in the incorrect arm helps, but a better solution is to use a complex water maze where turning bias is not an issue because the number of choice points dilutes turning bias background.

For passive avoidance, there are also concerns. First, is that the dependent measure is not an action but the absence of an action, i.e., the rat not crossing to the dark side. Rats may remain on the lighted side for many reasons, only one of which is related to memory. Other factors may be fear, heightened shock reactivity, hypoactivity, or failure to notice the divider door had opened. Passive avoidance data are highly variable, making error variance an issue that compromises test sensitivity. Trials to criterion compared with one-trial methods are better, but the data are still variable. If stress is a concern, foot-shock is the most stressful motivator of all. If there is a task where stress may interact with an experimental treatment, passive avoidance poses a greater risk confounding than mazes. Additionally, shock tasks need to include a test of shock threshold to ensure equal reactivity across groups, yet this is virtually never done in guideline studies. If the chemical changes shock thresholds, it is not possible to determine if a retention deficit is from impaired recall or a change in pain threshold. For these and other reasons the fact is that passive avoidance is insensitive. We tested rats in passive avoidance after developmental methamphetamine treatment. Developmental methamphetamine reliably impairs CWM and MWM performance, but passive avoidance is unaffected (Jablonski et al., 2015, 2017). Other tasks with some issues include appetitive RAM, spontaneous alternation, and novel object recognition. These tests can be used if done carefully but are not as robust as water mazes. One caution attends novel object recognition. If not carefully configured and test conditions not tightly controlled, the test is prone to variable results and for that reason may not be well-suited to regulatory studies.

The goal is to improve DNT and ANT test batteries. This should be a high priority to facilitate better translation of rodent data to humans. The focus should be on tests that assess homologous brain/memory systems across species. The field should abandon approaches and test ages because they are in guidelines written decades ago. Labs should urge regulators to accept better approaches and incorporate structure/function tests from above and seek regulatory permission to use them. It is time to rethink what is being done and use the epidemiological data from developmental studies in children from the past 40 years to improve animal guideline studies for safety assessment.

While the approach for drugs and food additives from FDA, EFSA (European Food Safety Authority), and EMA (European Medicines Agency) are different from those for pesticides and other chemicals under the jurisdiction of the EPA and OECD, all rely on animal data to assess hazard, and all need updating while high-throughput methods are under development. For the foreseeable future, rodent studies are the primary method of predicting human risk. Next in line are species such as the zebrafish; zebrafish can fill an important gap in the process by fitting between *in vitro* and rodent studies and thereby reduce the use of rodents. In the meantime, rat studies for DNT should not wait to be updated. We recommend that this be done using human neurodevelopmental epidemiological data to guide the choice of tests of higher cognitive functions. An example of what a cross-species set of tests might be like from children to rats to zebrafish is illustrated in Table 1.

**Table 1:** Human to rat to zebrafish tests assessing homologous functions.

**Function**	Human^*^	**Rat**	**Zebrafish**
Working memory	WISC-IV digit span Letter-number sequencing	Radial-arm maze Radial swimming maze	Three-chamber apparatus: Spatial alternation
Spatial memory	Virtual morris maze	Morris water maze (MWM)	Three-chamber apparatus: Spatial discrimination
Visual-spatial processing	NEPSY-II route finding, arrows WISC-V block design, visual puzzles	Cincinnati water maze	___
Motoric	NEPSY-II (Neuropsychological assessment battery) visuomotor precision BOT-2 (Bruininks-oseretsky test of motor proficiency)	Rotorod	Tap startle response
Executive functions	BRIEF-2 (Behavior rating inventory of executive function)	MWM reversal and shift	Three-chamber apparatus: reversal learning
Hyperactivity	BASC-3 (Behavioral assessment system for children)	Open-field	Open tank Swimming speed
Attention	Conner's CPT-II TOVA (Test of variables of attention)	Prepulse inhibition of startle 5-Choice Serial Reaction Time Test	___
Anxiety	Screen for child anxiety related disorders (SCARED)	Elevated plus maze Elevated zero maze	Novel tank diving test
Fear	Laboratory temperament assessment battery (Lab-TAB)	Conditioned freezing Novelty suppressed feeding	Predator avoidance test

* *We thank Kimberly Yolton, Ph.D., Professor of Pediatrics, University of Cincinnati College of Medicine and Cincinnati Children's Hospital Medical Center for the human test equivalencies*.

## Conclusions

The future of neurotoxicology involves two areas: those for regulatory studies and those for basic research. Changes are needed for both areas but are most needed in regulatory neurotoxicity because current approaches are obsolete. The guidelines for ANT and DNT by EPA have not been significantly updated in >30 years and this has resulted in suboptimal data being submitted to the Agency. Therefore, changes are overdue at EPA and OECD (Makris and Vorhees, 2015; Vorhees and Makris, 2015). Unfortunately, the changes that are made by OECD with endorsement by EPA do not improve cognitive assessment, the area the human data show is the most often adversely affected. In fact, in the extended one-generation reproductive toxicity guidelines from OECD cognitive testing has been eliminated (Beekhuijzen et al., 2016) and this protocol is intended to replace the use of separate guidelines for developmental, reproductive, general, and neurotoxicity by rolling them into one large “everything” study. This represents a disconnect between human data in children and the goal of improving regulatory guidelines aimed at protecting children.

This dichotomy is moving regulatory guidelines in the wrong direction, reducing emphasis on neurocognitive assessments in contradistinction to the data from children harmed by compounds such as lead, and many others cited above. It runs contrary to what is known about the inherent vulnerability of the brain during development, something that has been firmly established by 50 years of research. Instead, cost and time considerations dominate deliberations about how to revise existing guidelines.

Another need is more attention to high throughput screening methods that rely on species such as zebrafish. We illustrated the wide range of attributes of zebrafish that make them a valuable tool for screening and basic research. The value of this model is increasingly recognized, and they should play a role in ANT and DNT screening.

The future of basic neurotoxicological research, we suggest, would benefit from a core set of standard behavioral methods that should also align with the findings from human studies, because they would facilitate investigations of the cellular and molecular mechanisms that underlie behavioral findings. At present, studies showing behavioral and cellular effects tend to be correlational and often these correlations are low because they are non-specific. Advances in molecular genetic tools will help, but help would also accrue to using tests that map to brain regions and circuits that mediate the behavioral effect observed in rats, e.g., MWM deficits and hippocampal/parahippocampal damage. As such research progresses, improved brain regional molecular and behavioral relationships will facilitate the development of AOPs suitable for use in high throughput *in vitro* systems. Hence, if the learning tests we suggest were incorporated, the cellular basis for behavioral effects could be advanced since effects on these mazes point toward the brain regions and pathways likely to be affected.

The future of neurotoxicology, lies in methods better aligned to human findings. We offer here our view on how this might be done in the hope that discussion of where the field should go will continue.

## Author Contributions

All authors listed have made a substantial, direct and intellectual contribution to the work, and approved it for publication.

## Conflict of Interest

The authors declare that the research was conducted in the absence of any commercial or financial relationships that could be construed as a potential conflict of interest.

## References

[B1] Abu-Taweel G. M. Ajarem J. S. Ahmad M. (2013). Protective effect of curcumin on anxiety, learning behavior, neuromuscular activities, brain neurotransmitters and oxidative stress enzymes in cadmium intoxicated mice. J.Behav.Brain Sci. 3:74. 10.4236/jbbs.2013.31008

[B2] Aldridge J. E. Meyer A. Seidler F. J. Slotkin T. A. (2005). Developmental exposure to terbutaline and chlorpyrifos: pharmacotherapy of preterm labor and an environmental neurotoxicant converge on serotonergic systems in neonatal rat brain regions. *Toxicol. Appl*. Pharmacol. 203, 132–144. 10.1016/j.taap.2004.08.002 15710174

[B3] Ali S. Champagne D. L. Richardson M. K. (2012). Behavioral profiling of zebrafish embryos exposed to a panel of 60 water-soluble compounds. *Behav*. Brain Res. 228, 272–283. 10.1016/j.bbr.2011.11.020 22138507

[B4] Arthur D. Levin E. D. (2001). Spatial and non-spatial visual discrimination learning in zebrafish (Danio rerio). Animal Cog. 4, 125–131. 10.1007/s100710100111

[B5] Baiamonte M. Parker M. O. Vinson G. P. Brennan C. H. (2016). Sustained effects of developmental exposure to ethanol on zebrafish anxiety-like behaviour. PLoS ONE 11:e0148425. 10.1371/journal.pone.0148425 26862749 PMC4749633

[B6] Bailey G. P. Wise L. D. Buschmann J. Hurtt M. Fisher J. E. (2009). Pre- and postnatal developmental toxicity study design for pharmaceuticals. *Birth Defects Res. B Dev. Reprod*. Toxicol. 86, 437–445. 10.1002/bdrb.20217 20025040

[B7] Bailey J. Oliveri A. Levin E. D. (2013). Zebrafish model systems for developmental neurobehavioral toxicology. Birth Defects Res. 99, 14–23. 10.1002/bdrc.21027 23723169 PMC4000730

[B8] Bailey J. M. Oliveri A. N. Zhang C. Frazier J. M. Mackinnon S. Cole G. J.. (2015). Long-term behavioral impairment following acute embryonic ethanol exposure in zebrafish. *Neurotoxicol*. Teratol. 48, 1–8. 10.1016/j.ntt.2015.01.005 PMC4363207 25599606

[B9] Bailey K. R. Crawley J. N. (2009). “Chapter 5: anxiety-related behaviors,” in mice. in *Methods of Behavior Analysis in Neuroscience*. ed J. J. Buccafusco (Boca Raton, FL: CRC Press/Taylor & Francis).

[B10] Bal-Price A. Hogberg H. T. Crofton K. M. Daneshian M. FitzGerald R. E. Fritsche E.. (2018). Recommendation on test readiness criteria for new approach methods in toxicology: exemplified for developmental neurotoxicity. ALTEX 35, 306–352. 10.14573/altex.1712081 29485663 PMC6545888

[B11] Barron S. Hawkey A. Fields L. Littleton J. M. (2016). Animal models for medication development and application to treat fetal alcohol effects. *Int. Rev*. Neurobiol. 126, 423–440. 10.1016/bs.irn.2016.02.002 27055621

[B12] Basha C. C. Reddy R. G. (2015). Long-term changes in brain cholinergic system and behavior in rats following gestational exposure to lead: protective effect of calcium supplement. Interdiscipl.Toxicol. 8, 159–168. 10.1515/intox-2015-0025 27486377 PMC4961914

[B13] Baumann O. Mattingley J. B. (2010). Medial parietal cortex encodes perceived heading direction in humans. *J*. Neurosci. 30, 12897–12901. 10.1523/JNEUROSCI.3077-10.2010 PMC6633521 20881108

[B14] Beekhuijzen M. Barentsen H. Marsden E. Zmarowski A. Aujoulat M. Picut C.. (2016). Implementing the extended one-generation reproductive toxicity study (EOGRTS): important points to consider. *Crit. Rev*. Toxicol. 46, 332–347. 10.3109/10408444.2015.1137863 26941129

[B15] Blanchard R. J. Wall P. M. Blanchard D. C. (2003). Problems in the study of rodent aggression. *Horm*. Behav. 44, 161–170. 10.1016/S0018-506X(03)00127-2 14609538

[B16] Blaser R. E. Rosemberg D. B. (2012). Measures of anxiety in zebrafish (Danio rerio): dissociation of black/white preference and novel tank test. PLoS ONE 7:e36931. 10.1371/journal.pone.0036931 22615849 PMC3355173

[B17] Bolon B. Bradley A. Butt M. Jensen K. Krinke G. Mellon R. D. (2011). Compilation of international regulatory guidance documents for neuropathology assessment during nonclinical general toxicity and specialized neurotoxicity studies. Toxicol. Pathol. 39, 92–96. 10.1177/0192623310385145 21119055

[B18] Botreau F. Gisquet-Verrier P. (2010). Re-thinking the role of the dorsal striatum in egocentric/response strategy. *Front. Behav*. Neurosci. 4:7. 10.3389/fnbeh.2010.00007 PMC2831625 20204137

[B19] Braun A. A. Amos-Kroohs R. M. Gutierrez A. Lundgren K. H. Seroogy K. B. Skelton M. R.. (2015). Dopamine depletion in either the dorsomedial or dorsolateral striatum impairs egocentric Cincinnati water maze performance while sparing allocentric Morris water maze learning. *Neurobiol. Learn*. Mem. 118, 55–63. 10.1016/j.nlm.2014.10.009 25451306 PMC4331240

[B20] Braun A. A. Amos-Kroohs R. M. Gutierrez A. Lundgren K. H. Seroogy K. B. Vorhees C. V.. (2016). 6-Hydroxydopamine-induced dopamine reductions in the nucleus accumbens, but not the medial prefrontal cortex, impair cincinnati water maze egocentric and morris water maze allocentric navigation in male sprague-dawley rats. Neurotox. Res. 30, 199–212. 10.1007/s12640-016-9616-6 27003940

[B21] Braun A. A. Graham D. L. Schaefer T. L. Vorhees C. V. Williams M. T. (2012). Dorsal striatal dopamine depletion impairs both allocentric and egocentric navigation in rats. *Neurobiol. Learn*. Mem. 97, 402–408. 10.1016/j.nlm.2012.03.004 PMC4131757 22465436

[B22] Brock O. Bakker J. Baum M. J. (2013). Assessment of urinary pheromone discrimination, partner preference, and mating behaviors in female mice. Methods Mol. Biol. 1068m 319–329. 10.1007/978-1-62703-619-1_24 24014373

[B23] Brown T. I. Carr V. A. LaRocque K. F. Favila S. E. Gordon A. M. Bowles B.. (2016). Prospective representation of navigational goals in the human hippocampus. Science 352, 1323–1326. 10.1126/science.aaf0784 27284194

[B24] Brown T. I. Whiteman A. S. Aselcioglu I. Stern C. E. (2014). Structural differences in hippocampal and prefrontal gray matter volume support flexible context-dependent navigation ability. *J*. Neurosci. 34, 2314–2320. 10.1523/JNEUROSCI.2202-13.2014 PMC3913873 24501370

[B25] Burton D. F. Zhang C. Boa-Amponsem O. Mackinnon S. Cole G. J. (2017). Long-term behavioral change as a result of acute ethanol exposure in zebrafish: evidence for a role for sonic hedgehog but not retinoic acid signaling. *Neurotoxicol*. Teratol. 61, 66–73. 10.1016/j.ntt.2017.01.006 PMC5453814 28223149

[B26] Bushnell P. J. Strupp B. J. (2009). “Chapter 7: assessing attention in rodents,” in Methods of Behavior Analysis in Neuroscience, ed J. J. Buccafusco JJ (Boca Raton, FL: CRC Press/Taylor & Francis).

[B27] Buzsaki G. Moser E. I. (2013). Memory, navigation and theta rhythm in the hippocampal-entorhinal system. Nat. Neurosci. 16, 130–138. 10.1038/nn.3304 23354386 PMC4079500

[B28] Caito S. Aschner M. (2017). Developmental neurotoxicity of lead. Adv Neurobiol. 18, 3–12. 10.1007/978-3-319-60189-2_1 28889260

[B29] Cao F. Souders C. L. Li P. Pang S. Qiu L. Martyniuk C. J. (2018). Biological impacts of organophosphates chlorpyrifos and diazinon on development, mitochondrial bioenergetics, and locomotor activity in zebrafish (Danio rerio). *Neurotoxicol*. Teratol. 70, 18–27. 10.1016/j.ntt.2018.10.001 30290195

[B30] Cappon G. D. Bailey G. P. Buschmann J. Feuston M. H. Fisher J. E. Hew K. W.. (2009). Juvenile animal toxicity study designs to support pediatric drug development. *Birth Defects Res. B Dev. Reprod*. Toxicol. 86, 463–469. 10.1002/bdrb.20220 20025047

[B31] Carman H. M. Booze R. M. Mactutus C. F. (2002). Long-term retention of spatial navigation by preweanling rats. Dev. Psychobiol. 40, 68–77. 10.1002/dev.10014 11835152

[B32] Chen C. Tang Y. Jiang X. Qi Y. Cheng S. Qiu C.. (2012a). Early postnatal benzo(a)pyrene exposure in sprague-dawley rats causes persistent neurobehavioral impairments that emerge postnatally and continue into adolescence and adulthood. Toxicol. Sci. 125, 248–261. 10.1093/toxsci/kfr265 21984485

[B33] Chen J. Chen Y. Liu W. Bai C. Liu X. Liu K.. (2012b). Developmental lead acetate exposure induces embryonic toxicity and memory deficit in adult zebrafish. Neurotoxicol. Teratol. 34, 581–586. 10.1016/j.ntt.2012.09.001 22975620

[B34] Cornwell B. R. Johnson L. L. Holroyd T. Carver F. W. Grillon C. (2008). Human hippocampal and parahippocampal theta during goal-directed spatial navigation predicts performance on a virtual morris water maze. *J*. Neurosci. 28, 5983–5990. 10.1523/JNEUROSCI.5001-07.2008 PMC2584780 18524903

[B35] Cory-Slechta D. A. Crofton K. M. Foran J. A. Ross J. F. Sheets L. P. Weiss B.. (2001). Methods to identify and characterize developmental neurotoxicity for human health risk assessment. I: behavioral effects. Environ. Health Perspect. 109, 79–91. 10.1289/ehp.01109s179 11250808 PMC1240545

[B36] Cullen K. E. Taube J. S. (2017). Our sense of direction: progress, controversies and challenges. Nat. Neurosci. 20, 1465–1473. 10.1038/nn.4658 29073639 PMC10278035

[B37] Cutler A. R. Wilkerson A. E. Gingras J. L. Levin E. D. (1996). Prenatal cocaine and/or nicotine exposure in rats: preliminary findings on long-term cognitive outcome and genital development at birth. Neurotoxicol. Teratol. 18, 635–643. 10.1016/S0892-0362(96)00125-0 8947940

[B38] DeCoteau W. E. Hoang L. Huff L. Stone A. Kesner R. P. (2004). Effects of hippocampus and medial caudate nucleus lesions on memory for direction information in rats. Behav. Neurosci. 118, 540–545. 10.1037/0735-7044.118.3.540 15174931

[B39] Delcasso S. Huh N. Byeon J. S. Lee J. Jung M. W. Lee I. (2014). Functional relationships between the hippocampus and dorsomedial striatum in learning a visual scene-based memory task in rats. *J*. Neurosci. 34, 15534–15547. 10.1523/JNEUROSCI.0622-14.2014 PMC4236391 25411483

[B40] Delis F. Polissidis A. Poulia N. Justinova Z. Nomikos G. G. Goldberg S. R.. (2016). Attenuation of cocaine-induced conditioned place preference and motor activity via cannabinoid CB2 receptor agonism and CB1 receptor antagonism in rats. Int. J. Neuropsychopharmacol. 20, 269–278. 10.1093/ijnp/pyw102 27994006 PMC5408977

[B41] Dudchenko P. A. (2004). An overview of the tasks used to test working memory in rodents. *Neurosci. Biobehav*. Rev. 28, 699–709. 10.1016/j.neubiorev.2004.09.002 15555679

[B42] Eddins D. Cerutti D. Williams P. Linney E. Levin E. D. (2010). Zebrafish provide a sensitive model of persisting neurobehavioral effects of developmental chlorpyrifos exposure: comparison with nicotine and pilocarpine effects and relationship to dopamine deficits. Neurotoxicol. Teratol. 32, 99–108. 10.1016/j.ntt.2009.02.005 19268529 PMC2885770

[B43] Ernst M. Moolchan E. T. Robinson M. L. (2001). Behavioral and neural consequences of prenatal exposure to nicotine. *J. Am. Acad. Child Adolesc*. Psychiatry 40, 630–641. 10.1097/00004583-200106000-00007 11392340

[B44] Ferbinteanu J. (2016). Contributions of hippocampus and striatum to memory-guided behavior depend on past experience. J. Neurosci. 36, 6459–6470. 10.1523/JNEUROSCI.0840-16.2016 27307234 PMC5015782

[B45] Ferbinteanu J. (2020). The hippocampus and dorsolateral striatum integrate distinct types of memories through time and space, respectively. *J*. Neurosci. 40, 9055–9065. 10.1523/JNEUROSCI.1084-20.2020 PMC7673003 33051349

[B46] Fernandes Y. Buckley D. M. Eberhart J. K. (2018). Diving into the world of alcohol teratogenesis: a review of zebrafish models of fetal alcohol spectrum disorder. Biochem. Cell Biol. 96, 88–97. 10.1139/bcb-2017-0122 28817785 PMC7413215

[B47] Fernandes Y. Gerlai R. (2009). Long-term behavioral changes in response to early developmental exposure to ethanol in zebrafish. *Alcohol. Clin. Exp*. Res. 33, 601–609. 10.1111/j.1530-0277.2008.00874.x PMC2715552 19183139

[B48] File S. E. Seth P. (2003). A review of 25 years of the social interaction test. Eur. J. Pharmacol. 463, 35–53. 10.1016/S0014-2999(03)01273-1 12600701

[B49] Fisher J. E. Jr. Ravindran A. Elayan I. (2019). CDER experience with juvenile animal studies for CNS drugs. Int. J. Toxicol. 38, 88–95. 10.1177/1091581818824313 30739550

[B50] Foster P. M. (2014). Regulatory Forum opinion piece: new testing paradigms for reproductive and developmental toxicity–the NTP modified one generation study and OECD 443. *Toxicol*. Pathol. 42, 1165–1167. 10.1177/0192623314534920 PMC4245367 24862797

[B51] Fritsche E. Grandjean P. Crofton K. M. Aschner M. Goldberg A. Heinonen T.. (2018). Consensus statement on the need for innovation, transition and implementation of developmental neurotoxicity (DNT) testing for regulatory purposes. *Toxicol. Appl*. Pharmacol. 354, 3–6. 10.1016/j.taap.2018.02.004 PMC6097873 29447839

[B52] Gauthier P. T. Holloway A. C. Vijayan M. M. (2020). Vape flavourants dull sensory perception and cause hyperactivity in developing zebrafish embryos. Biol. Lett. 16:20200361. 10.1098/rsbl.2020.0361 32961088 PMC7532713

[B53] Grandjean P. Landrigan P. J. (2006). Developmental neurotoxicity of industrial chemicals. Lancet 368, 2167–2178. 10.1016/S0140-6736(06)69665-7 17174709

[B54] Grandjean P. Landrigan P. J. (2014). Neurobehavioural effects of developmental toxicity. Lancet Neurol. 13, 330–338. 10.1016/S1474-4422(13)70278-3 24556010 PMC4418502

[B55] Green J. T. Tran T. Steinmetz J. E. Goodlett C. R. (2002). Neonatal ethanol produces cerebellar deep nuclear cell loss and correlated disruption of eyeblink conditioning in adult rats. Brain Res. 956, 302–311. 10.1016/S0006-8993(02)03561-8 12445699

[B56] Hall B. J. Cauley M. Burke D. A. Kiany A. Slotkin T. A. Levin E. D. (2016). Cognitive and behavioral impairments evoked by low-level exposure to tobacco smoke components: comparison with nicotine alone. *Toxicol*. Sci. 151, 236–244. 10.1093/toxsci/kfw042 PMC4880133 26919958

[B57] Hartley T. Maguire E. A. Spiers H. J. Burgess N. (2003). The well-worn route and the path less traveled: distinct neural bases of route following and wayfinding in humans. Neuron 37, 877–888. 10.1016/S0896-6273(03)00095-3 12628177

[B58] Hatherell S. Baltazar M. T. Reynolds J. Carmichael P. L. Dent M. Li H.. (2020). Identifying and characterizing stress pathways of concern for consumer safety in next-generation risk assessment. *Toxicol*. Sci. 176, 11–33. 10.1093/toxsci/kfaa054 PMC7357173 32374857

[B59] Hawkey A. Junaid S. Yao L. Spiera Z. White H. Cauley M.. (2019). Gestational exposure to nicotine and/or benzo[a]pyrene causes long-lasting neurobehavioral consequences. Birth Defects Res. 111, 1248–1258. 10.1002/bdr2.1568 31368242 PMC6983916

[B60] Hawkey A. B. Glazer L. Dean C. Wells C. N. Odamah K. A. Slotkin T. A.. (2020). Adult exposure to insecticides causes persistent behavioral and neurochemical alterations in zebrafish. Neurotoxicol. Teratol. 78:106853. 10.1016/j.ntt.2019.106853 31911208 PMC7061078

[B61] Heyer D. B. Meredith R. M. (2017). Environmental toxicology: sensitive periods of development and neurodevelopmental disorders. Neurotoxicology 58, 23–41. 10.1016/j.neuro.2016.10.017 27825840

[B62] Holden L. L. Truong L. Simonich M. T. Tanguay R. L. (2020). Assessing the hazard of E-Cigarette flavor mixtures using zebrafish. *Food Chem*. Toxicol. 136:110945. 10.1016/j.fct.2019.110945 PMC7811276 31712102

[B63] Icenogle L. M. Christopher N. C. Blackwelder W. P. Caldwell D. P. Qiao D. Seidler F. J.. (2004). Behavioral alterations in adolescent and adult rats caused by a brief subtoxic exposure to chlorpyrifos during neurulation. *Neurotoxicol*. Teratol. 26, 95–101. 10.1016/j.ntt.2003.09.001 15001218

[B64] Jablonski S. A. Williams M. T. Vorhees C. V. (2015). Neurobehavioral effects from developmental methamphetamine exposure. Curr. Top. Behav. Neurosci. 29, 183–230. 10.1007/7854_2015_405 26520437

[B65] Jablonski S. A. Williams M. T. Vorhees C. V. (2017). Learning and memory effects of neonatal methamphetamine exposure in rats: role of reactive oxygen species and age at assessment. Synapse 71. 10.1002/syn.21992 28686793

[B66] Jog M. S. Kubota Y. Connolly C. I. Hillegaart V. Graybiel A. M. (1999). Building neural representations of habits. Science 286, 1745–1749. 10.1126/science.286.5445.1745 10576743

[B67] Kaidanovich-Beilin O. Lipina T. Vukobradovic I. Roder J. Woodgett J. R. (2011). Assessment of social interaction behaviors. J. Vis. Exp. 48:2473. 10.3791/2473 PMC3197404 21403628

[B68] Kant G. J. Yen M. H. D'Angelo P. C. Brown A. J. Eggleston T. (1988). Maze performance: a direct comparison of food vs. water mazes. *Pharmacol. Biochem*. Behav. 31, 487–491. 10.1016/0091-3057(88)90378-4 2854267

[B69] Karri V. Schuhmacher M. Kumar V. (2016). Heavy metals (Pb, Cd, As and MeHg) as risk factors for cognitive dysfunction: a general review of metal mixture mechanism in brain. Environ. Toxicol. Pharmacol. 48, 203–213. 10.1016/j.etap.2016.09.016 27816841

[B70] Knecht A. L. Truong L. Simonich M. T. Tanguay R. L. (2017). Developmental benzo[a]pyrene (B[a]P) exposure impacts larval behavior and impairs adult learning in zebrafish. *Neurotoxicol*. Teratol. 59, 27–34. 10.1016/j.ntt.2016.10.006 PMC5235990 27989697

[B71] Ladu F. Bartolini T. Panitz S. G. Chiarotti F. Butail S. Macri S.. (2015). Live predators, robots, and computer-animated images elicit differential avoidance responses in zebrafish. Zebrafish 12, 205–214. 10.1089/zeb.2014.1041 25734228

[B72] Langheinrich U. (2003). Zebrafish: a new model on the pharmaceutical catwalk. BioEssays 25, 904–912. 10.1002/bies.10326 12938180

[B73] Lanzoni A. Castoldi A. F. Kass G. E. Terron A. De Seze G. Bal-Price A.. (2019). Advancing human health risk assessment. EFSA J. 17:e170712. 10.2903/j.efsa.2019.e170712 32626449 PMC7015480

[B74] Lee J. Freeman J. L. (2014). Zebrafish as a model for investigating developmental lead (Pb) neurotoxicity as a risk factor in adult neurodegenerative disease: a mini-review. Neurotoxicology 43, 57–64. 10.1016/j.neuro.2014.03.008 24698670

[B75] Levin E. D. (2011). Zebrafish assessment of cognitive improvement and anxiolysis: filling the gap between *in vitro* and rodent models for drug development. Rev. Neurosci. 22, 75–84. 10.1515/rns.2011.009 21615262 PMC4691346

[B76] Levin E. D. Addy N. Baruah A. Elias A. Christopher N. C. Seidler F. J.. (2002). Prenatal chlorpyrifos exposure in rats causes persistent behavioral alterations. Neurotoxicol. Teratol. 24, 733–741. 10.1016/S0892-0362(02)00272-6 12460655

[B77] Levin E. D. Addy N. Nakajima A. Christopher N. C. Seidler F. J. Slotkin T. A. (2001). Persistent behavioral consequences of neonatal chlorpyrifos exposure in rats. Brain Res. Dev. Brain Res. 130, 83–89. 10.1016/S0165-3806(01)00215-2 11557096

[B78] Levin E. D. Chrysanthis E. Yacisin K. Linney E. (2003). Chlorpyrifos exposure of developing zebrafish: effects on survival and long-term effects on response latency and spatial discrimination. *Neurotoxicol*. Teratol. 25, 51–57. 10.1016/S0892-0362(02)00322-7 12633736

[B79] Levin E. D. Lawrence S. Petro A. Horton K. Seidler F. J. Slotkin T. A. (2006). Increased nicotine self-administration following prenatal exposure in female rats. *Pharmacol. Biochem*. Behav. 85, 669–674. 10.1016/j.pbb.2006.11.006 PMC1797890 17196243

[B80] Levin E. D. Swain H. A. Donerly S. Linney E. (2004). Developmental chlorpyrifos effects on hatchling zebrafish swimming behavior. Neurotoxicol. Teratol. 26, 719–723. 10.1016/j.ntt.2004.06.013 15451035

[B81] Lopez-Schier H. (2019). Neuroplasticity in the acoustic startle reflex in larval zebrafish. *Curr. Opin*. Neurobiol. 54, 134–139. 10.1016/j.conb.2018.10.004 30359930

[B82] Luca R. M. Gerlai R. (2012). In search of optimal fear inducing stimuli: Differential behavioral responses to computer animated images in zebrafish. Behav. Brain Res. 226, 66–76. 10.1016/j.bbr.2011.09.001 21920389 PMC3203217

[B83] Lucena G. M. Porto F. A. Campos E. G. Azevedo M. S. Cechinel-Filho V. Prediger R. D.. (2010). Cipura paludosa attenuates long-term behavioral deficits in rats exposed to methylmercury during early development. *Ecotoxicol. Environ*. Saf. 73, 1150–1158. 10.1016/j.ecoenv.2010.04.008 20447691

[B84] Makris S. L. Raffaele K. Allen S. Bowers W. J. Hass U. Alleva E.. (2009). A retrospective performance assessment of the developmental neurotoxicity study in support of OECD test guideline 426. *Environ*. Health Perspect 117, 17–25. 10.1289/ehp.11447 PMC2627860 19165382

[B85] Makris S. L. Vorhees C. V. (2015). Assessment of learning, memory and attention in developmental neurotoxicity regulatory studies: introduction. *Neurotoxicol*. Teratol. 52, 62–67. 10.1016/j.ntt.2015.05.010 26049062

[B86] Marx-Stoelting P. Solano M. L. M. Aoyama H. Adams R. H. Bal-Price A. Buschmann J.. (2020). 25(th) anniversary of the Berlin Workshop on Developmental Toxicology: DevTox database update, challenges in risk assessment of developmental neurotoxicity and alternative methodologies in bone development and growth. Reprod. Toxicol. S0890-6238(20)30254-9. 10.1016/j.reprotox.2020.11.003 PMC9337270 33278556

[B87] Mathur P. Lau B. Guo S. (2011). Conditioned place preference behavior in zebrafish. Nat. Protoc. 6:338–345. 10.1038/nprot.2010.201 21372814 PMC6233885

[B88] Mattson S. N. Bernes G. A. Doyle L. R. (2019). Fetal alcohol spectrum disorders: a review of the neurobehavioral deficits associated with prenatal alcohol exposure. *Alcohol. Clin. Exp*. Res. 43, 1046–1062. 10.1111/acer.14040 PMC6551289 30964197

[B89] Nebert D. W. Ingelman-Sundberg M. (2016). What do animal experiments tell us that *in vitro* systems cannot? The human toxome project. *Regul. Toxicol*. Pharmacol. 75, 1–4. 10.1016/j.yrtph.2015.09.027 26409932

[B90] Ohta K. Sakata-Haga H. Fukui Y. (2012). Prenatal ethanol exposure impairs passive avoidance acquisition and enhances unconditioned freezing in rat offspring. Behav. Brain Res. 234, 255–258. 10.1016/j.bbr.2012.07.001 22776160

[B91] Padilla S. Corum D. Padnos B. Hunter D. L. Beam A. Houck K. A.. (2012). Zebrafish developmental screening of the ToxCast Phase I chemical library. *Reprod*. Toxicol. 33, 174–187. 10.1016/j.reprotox.2011.10.018 22182468

[B92] Paparella M. Bennekou S. H. Bal-Price A. (2020). An analysis of the limitations and uncertainties of *in vivo* developmental neurotoxicity testing and assessment to identify the potential for alternative approaches. *Reprod*. Toxicol. 96, 327–336. 10.1016/j.reprotox.2020.08.002 32781019

[B93] Parker B. Connaughton V. P. (2007). Effects of nicotine on growth and development in larval zebrafish. Zebrafish 4, 59–68. 10.1089/zeb.2006.9994 18041943

[B94] Pham M. Raymond J. Hester J. Kyzar E. Gaikwad S. Bruce I.. (2012). Assessing Social Behavior Phenotypes in Adult Zebrafish: Shoaling, Social Preference, and Mirror Biting Tests, Zebrafish Protocols for Neurobehavioral Research. Totowa, NJ: Humana Press. 10.1007/978-1-61779-597-8_17

[B95] Piersma A. H. Tonk E. C. Makris S. L. Crofton K. M. Dietert R. R. Van L. H. (2012). Juvenile toxicity testing protocols for chemicals. *Reprod*. Toxicol 34, 482–486. 10.1016/j.reprotox.2012.04.010 22564981

[B96] Pistollato F. de Gyves E. M. Carpi D. Bopp S. K. Nunes C. Worth A.. (2020). Assessment of developmental neurotoxicity induced by chemical mixtures using an adverse outcome pathway concept. *Environ*. Health 19:23. 10.1186/s12940-020-00578-x PMC7038628 32093744

[B97] Pitzer E. M. Sugimoto C. Gudelsky G. A. Huff Adams C. L. Williams M. T. Vorhees C. V. (2019). Deltamethrin exposure daily from postnatal day 3-20 in Sprague-Dawley rats causes long-term cognitive and behavioral deficits. *Toxicol*. Sci. 169, 511–523. 10.1093/toxsci/kfz067 PMC6542333 30850843

[B98] Porsolt R. D. Anton G. Blavet N. Jalfre M. (1978). Behavioural despair in rats: a new model sensitive to antidepressant treatments. Eur. J. Pharmacol. 47, 379–391. 10.1016/0014-2999(78)90118-8 204499

[B99] Qin M. Wong A. Seguin D. Gerlai R. (2014). Induction of social behavior in zebrafish: live versus computer animated fish as stimuli. Zebrafish 11, 185–197. 10.1089/zeb.2013.0969 24575942 PMC4050712

[B100] Regan S. L. Sugimoto C. Dawson H. E. Tempe E. A. Lingo A. N. Williams M. T.. (2020). Comparison of constitutive Lphn3 KO and Lphn3-TH-Cre conditional KO Sprague-Dawley rats on activity, learning, and memory. Neurotoxicol. Teratol. 79:106885.32298771

[B101] Ricceri L. Markina N. Valanzano A. Fortuna S. Cometa M. F. Meneguz A.. (2003). Developmental exposure to chlorpyrifos alters reactivity to environmental and social cues in adolescent mice. *Toxicol. Appl*. Pharmacol. 191, 189–201. 10.1016/S0041-008X(03)00229-1 13678652

[B102] Ricceri L. Venerosi A. Capone F. Cometa M. F. Lorenzini P. Fortuna S.. (2006). Developmental neurotoxicity of organophosphorous pesticides: fetal and neonatal exposure to chlorpyrifos alters sex-specific behaviors at adulthood in mice. Toxicol. Sci. 93, 105–113. 10.1093/toxsci/kfl032 16760416

[B103] Rice C. Ghorai J. K. Zalewski K. Weber D. N. (2011). Developmental lead exposure causes startle response deficits in zebrafish. *Aquat*. Toxicol. 105, 600–608. 10.1016/j.aquatox.2011.08.014 PMC3207002 21955963

[B104] Roegge C. S. Timofeeva O. A. Seidler F. J. Slotkin T. A. Levin E. D. (2008). Developmental diazinon neurotoxicity in rats: later effects on emotional response. *Brain Res*. Bull. 75, 166–172. 10.1016/j.brainresbull.2007.08.008 PMC2253685 18158111

[B105] Rudy J. W. Paylor R. (1988). Reducing the temporal demands of the Morris place-learning task fails to ameliorate the place-learning impairment of preweanling rats. Psychobiology 16, 152–156.

[B106] Rudy J. W. Stadler-Morris S. Albert P. (1987). Ontogeny of spatial navigation behaviors in the rat: dissociation of “proximal” and “distal” -cue-based behaviors. Behav. Neurosci. 101, 62–73. 10.1037/0735-7044.101.1.62 3828056

[B107] Sanders A. P. Claus Henn B. Wright R. O. (2015). Perinatal and childhood exposure to cadmium, manganese, and metal mixtures and effects on cognition and behavior: a review of recent literature. Curr Environ. Health Rep. 2, 284–294. 10.1007/s40572-015-0058-8 26231505 PMC4531257

[B108] Santos J. M. Gargaro A. C. Oliveira A. R. Masson S. Brandao M. L. (2005). Pharmacological dissociation of moderate and high contextual fear as assessed by freezing behavior and fear-potentiated startle. Eur. Neuropsychopharmacol. 15, 239–246. 10.1016/j.euroneuro.2004.10.004 15695072

[B109] Schenk F. (1985). Development of place navigation in rats from weaning to puberty. *Behav*. Neural. Biol. 43, 69–85. 10.1016/S0163-1047(85)91510-9 3994625

[B110] Schneider T. Ilott N. Brolese G. Bizarro L. Asherson P. J. Stolerman I. P. (2011). Prenatal exposure to nicotine impairs performance of the 5-choice serial reaction time task in adult rats. Neuropsychopharmacology 36, 1114–1125. 10.1038/npp.2010.249 21289608 PMC3077278

[B111] Skelton M. R. Williams M. T. Schaefer T. L. Vorhees C. V. (2007). Neonatal (+)-methamphetamine increases brain derived neurotrophic factor, but not nerve growth factor, during treatment and results in long-term spatial learning deficits. Psychoneuroendocrinology 32, 734–745. 10.1016/j.psyneuen.2007.05.004 17606327 PMC2756096

[B112] Sledge D. Yen J. Morton T. Dishaw L. Petro A. Donerly S.. (2011). Critical duration of exposure for developmental chlorpyrifos-induced neurobehavioral toxicity. *Neurotoxicol*. Teratol. 33, 742–751. 10.1016/j.ntt.2011.06.005 PMC3225562 21745564

[B113] Smith L. E. Carvan M. J. Dellinger J. A. Ghorai J. K. White D. B. Williams F. E.. (2010). Developmental selenomethionine and methylmercury exposures affect zebrafish learning. *Neurotoxicol*. Teratol. 32, 246–255. 10.1016/j.ntt.2009.09.004 PMC2838996 19800969

[B114] Sobotka T. J. Ekelman K. B. Slikker W. Jr. Raffaele K. Hattan D. G. (1996). Food and drug administration proposed guidelines for neurotoxicological testing of food chemicals. Neurotoxicology 17, 825–836. 9086506

[B115] Speedie N. Gerlai R. (2008). Alarm substance induced behavioral responses in zebrafish (Danio rerio). *Behav*. Brain Res. 188, 168–177. 10.1016/j.bbr.2007.10.031 PMC2715551 18054804

[B116] Stephens M. L. (2009). Personal reflections on Russell and Burch, FRAME, and the HSUS. Altern Lab Anim. 37, 29–33. 10.1177/026119290903702S21 20105008

[B117] Stewart A. Maximino C. Brito T. M. Herculano Gouveia A. M. A. Morato S. Cahat J. M.. (2011). Neurophenotyping of Adult Zebrafish Using the Light/Dark Box Paradigm, Zebrafish Neurobehavioral Protocols. New York, NY: Humana Press. 10.1007/978-1-60761-953-6_13

[B118] Stewart A. M. Grossman L. Collier A. D. Echevarria D. J. Kalueff A. V. (2015). Anxiogenic-like effects of chronic nicotine exposure in zebrafish. Pharmacol. Biochem. Behav. 139, 112–120. 10.1016/j.pbb.2015.01.016 25643654

[B119] Strungaru S. A. Robea M. A. Plavan G. Todirascu-Ciornea E. Ciobica A. Nicoara M. (2018). Acute exposure to methylmercury chloride induces fast changes in swimming performance, cognitive processes and oxidative stress of zebrafish (Danio rerio) as reference model for fish community. *J. Trace Elem. Med*. Biol. 47, 115–123. 10.1016/j.jtemb.2018.01.019 29544797

[B120] Tait D. S. Chase E. A. Brown V. J. (2014). Attentional set-shifting in rodents: a review of behavioural methods and pharmacological results. *Curr. Pharm*. Des. 20, 5046–5059. 10.2174/1381612819666131216115802 24345263

[B121] Takao K. Miyakawa T. (2006). Light/dark transition test for mice. J. Vis. Exp. 104. 10.3791/104 18704188 PMC2504462

[B122] Talbot W. S. Hopkins N. (2000). Zebrafish mutations and functional analysis of the vertebrate genome. Genes Dev. 14, 755–762. 10766732

[B123] Tilton F. A. Bammler T. K. Gallagher E. P. (2011). Swimming impairment and acetylcholinesterase inhibition in zebrafish exposed to copper or chlorpyrifos separately, or as mixtures. *Comp. Biochem. Physiol. C Toxicol*. Pharmacol. 153, 9–16. 10.1016/j.cbpc.2010.07.008 PMC3034093 20692364

[B124] Timofeeva O. A. Roegge C. S. Seidler F. J. Slotkin T. A. Levin E. D. (2008). Persistent cognitive alterations in rats after early postnatal exposure to low doses of the organophosphate pesticide diazinon. Neurotoxicol. Teratol. 30, 38–45. 10.1016/j.ntt.2007.10.002 18096363 PMC2262840

[B125] Tonkiss J. Shultz P. Galler J. R. (1992). Long-Evans and Sprague-Dawley rats differ in their spatial navigation performance during ontogeny and at maturity. Dev. Psychobiol. 25, 567–579. 10.1002/dev.420250804 1487082

[B126] Tu H. Fan C. Chen X. Liu J. Wang B. Huang Z.. (2017). Effects of cadmium, manganese, and lead on locomotor activity and neurexin 2a expression in zebrafish. *Environ. Toxicol*. Chem. 36, 2147–2154. 10.1002/etc.3748 28120348

[B127] Vaglenova J. Birru S. Pandiella N. M. Breese C. R. (2004). An assessment of the long-term developmental and behavioral teratogenicity of prenatal nicotine exposure. *Behav*. Brain Res. 150, 159–170. 10.1016/j.bbr.2003.07.005 15033289

[B128] Venerosi A. Calamandrei G. Ricceri L. (2006). A social recognition test for female mice reveals behavioral effects of developmental chlorpyrifos exposure. *Neurotoxicol*. Teratol. 28, 466–471. 10.1016/j.ntt.2006.05.003 16814983

[B129] Vorhees C. V. He E. Skelton M. R. Graham D. L. Schaefer T. L. Grace C. E.. (2011). Comparison of (+)-methamphetamine, +/–methylenedioxymethamphetamine, (+)-amphetamine and +/–fenfluramine in rats on egocentric learning in the Cincinnati water maze. Synapse 65, 368–378. 10.1002/syn.20854 20730798 PMC2994999

[B130] Vorhees C. V. Makris S. L. (2015). Assessment of learning, memory, and attention in developmental neurotoxicity regulatory studies: synthesis, commentary, and recommendations. Neurotoxicol. Teratol. 52, 109–115. 10.1016/j.ntt.2015.10.004 26526903

[B131] Vorhees C. V. Sprowles J. N. Regan S. L. Williams M. T. (2018). A better approach to *in vivo* developmental neurotoxicity assessment: alignment of rodent testing with effects seen in children after neurotoxic exposures. *Toxicol. Appl*. Pharmacol. 354, 176–190. 10.1016/j.taap.2018.03.012 29544898

[B132] Vorhees C. V. Williams M. T. (2014). Assessing spatial learning and memory in rodents. ILAR J. 55, 310–332. 10.1093/ilar/ilu013 25225309 PMC4240437

[B133] Vorhees C. V. Williams M. T. (2016). Cincinnati water maze: a review of the development, methods, and evidence as a test of egocentric learning and memory. *Neurotoxicol*. Teratol. 57, 1–19. 10.1016/j.ntt.2016.08.002 PMC5056837 27545092

[B134] Walf A. A. Frye C. A. (2007). The use of the elevated plus maze as an assay of anxiety-related behavior in rodents. Nat. Protoc. 2, 322–328. 10.1038/nprot.2007.44 17406592 PMC3623971

[B135] Weber D. N. Ghorai J. K. (2013). Experimental design affects social behavior outcomes in adult zebrafish developmentally exposed to lead. Zebrafish 10, 294–302. 10.1089/zeb.2012.0780 23672286

[B136] Weston H. I. Weston D. D. Allen J. L. Cory-Slechta D. A. (2014). Sex-dependent impacts of low-level lead exposure and prenatal stress on impulsive choice behavior and associated biochemical and neurochemical manifestations. Neurotoxicology 44, 169–183. 10.1016/j.neuro.2014.06.013 25010656 PMC4175053

[B137] Wetmore B. A. Wambaugh J. F. Allen B. Ferguson S. S. Sochaski M. A. Setzer R. W.. (2015). Incorporating high-throughput exposure predictions with dosimetry-adjusted *in vitro* bioactivity to inform chemical toxicity testing. *Toxicol*. Sci. 148, 121–136. 10.1093/toxsci/kfv171 PMC4620046 26251325

[B138] Whishaw I. Q. Maaswinkel H. (1998). Rats with fimbria-fornix lesions are impaired in path integration: a role for the hippocampus in “sense of direction”. J. Neurosci. 18, 3050–3058. 10.1523/JNEUROSCI.18-08-03050.1998 9526022 PMC6792581

[B139] Yang D. Lauridsen H. Buels K. Chi L. H. La Du J. Bruun D. A.. (2011). Chlorpyrifos-oxon disrupts zebrafish axonal growth and motor behavior. *Toxicol*. Sci. 121, 146–159. 10.1093/toxsci/kfr028 PMC3080187 21346248

[B140] Zeddies D. G. Fay R. R. (2005). Development of the acoustically evoked behavioral response in zebrafish to pure tones. J. Exp. Biol. 208, 1363–1372. 10.1242/jeb.01534 15781896

[B141] Zhang W. Tian F. Zheng J. Li S. Qiang M. (2016). Chronic administration of benzo(a)pyrene induces memory impairment and anxiety-like behavior and increases of NR2B DNA methylation. PLoS ONE 11:e0149574. 10.1371/journal.pone.0149574 26901155 PMC4768874

